# Oral health-related quality of life among chronic kidney disease patients across disease stages: A comparative analysis

**DOI:** 10.6026/973206300223142

**Published:** 2026-05-31

**Authors:** Sandhya Chavan, Roshni Dupare, Swapnil Singhai, Megha Goyal, Bhuvan Nagpal, Tamoghna Biswas

**Affiliations:** 1 Department of Public Health Dentistry, Government Dental College and Hospital, Mumbai, Maharashtra, India; 2 Department of Public Health Dentistry, Government Dental College and Hospital, Nagpur, Maharashtra, India; 3 Department of Oral and Maxillofacial Surgery, Bhagyodaya Tirth Hospital, Sagar, Madhya Pradesh, India; 4 Department of Oral Medicine and Radiology, Maharana Pratap College of Dentistry and Research Centre, Gwalior, Madhya Pradesh, India; 5 Department of Oral pathology and microbiology, Tohana Manglam Diagnostics and Centre for Oral Pathology & Maxillofacial Diagnostics, Hisar, Haryana, India; 6 Department of Oral Medicine and Radiology, Saraswati Dental College, Atal Bihari Vajpayee University, Lucknow, Uttar Pradesh, India

**Keywords:** Oral health-related quality of life (OHRQoL), chronic kidney disease (CKD), progressive systemic disorder

## Abstract

Chronic kidney disease (CKD) may worsen oral health and oral health-related quality of life (OHRQoL), yet stage-wise impact remains unclear. Therefore, it is of interest to compare OHRQoL among 100 CKD patients stratified by disease stage using estimated glomerular filtration rate (eGFR). Oral health status was assessed for caries, periodontal condition and xerostomia, while OHRQoL was measured using the Oral Health Impact Profile-14 (OHIP-14). Higher CKD stages showed significantly increased OHIP-14 scores with greater prevalence of xerostomia, periodontal disease and mucosal lesions. Thus, we show an association between CKD progression and declining OHRQoL, supporting early dental screening and integrated care.

## Background:

The persistent and irreversible kidney illness known as chronic kidney disease (CKD) causes a steady decline in kidney function over time. Because of its high morbidity, growing incidence and enormous financial impact on healthcare systems, it poses a serious threat to public health across the world. Cardiovascular disease, anemia, bone metabolism abnormalities, immunological dysfunction and other systemic consequences of chronic kidney disease (CKD) greatly diminish the quality of life for those afflicted. The prevalence of chronic kidney disease is on the rise owing to factors such as an aging population, an increase in the incidence of diabetes mellitus and hypertension and other medical conditions. Worldwide health estimates place the number of people afflicted by this condition at 8-16% of the worldwide population [1]. The estimated glomerular filtration rate (eGFR) is the standard method for categorizing chronic kidney disease (CKD). Stages 1 and 2 denote early disease, stage 3 moderate kidney impairment and stages 4 and 5 advanced kidney failure necessitating dialysis or renal replacement therapy [2]. Oral manifestations of systemic and metabolic abnormalities may occur in individuals as the illness advances. Periodontal disease, dental caries, changed taste sensation, delayed wound healing, xerostomia; uremic stomatitis, mucosal pallor and altered taste perception are common oral consequences [3]. Maintaining good nutrition, social functioning and general health all depend on good oral health. Oral health may have a major impact on the day-to-day functionality and mental health of individuals with chronic systemic diseases like CKD. Unfortunately, therapeutic therapy of chronic kidney disease (CKD) mostly addresses potentially fatal systemic consequences, which means that patients' oral health requirements are often neglected. Consequently, patients' quality of life may suffer as a result of the progressive worsening of untreated oral disorders [4]. The idea of oral health-related quality of life (OHRQoL) has become more popular in recent years. It's a multi-faceted way to quantify how good dental health affects a person's overall health, happiness and social life. One of the most popular ways to measure the impact of oral health issues on people's day-to-day lives, emotions and relationships is via an OHRQoL assessment instrument like the Oral Health Impact Profile (OHIP) questionnaire. Because they capture the patient's subjective assessment of their dental health, these measurements provide useful information beyond clinical markers [5]. Multiple studies have shown that overall health-related quality of life (OHRQoL) is much worse in those with chronic systemic disorders than in healthy people. Metabolic abnormalities, immunological suppression and medication-related oral problems put CKD patients at increased risk. The risk of periodontal disease and dental caries may be higher in those with uremia and diminished salivary flow and those on long-term dialysis may be even more vulnerable to infections because of the additional damage to oral tissues [6]. There is a two-way street between chronic kidney disease and dental health. Inflammation throughout the body may accelerate the course of kidney disease and poor dental hygiene, especially long-term periodontal infection, can exacerbate this problem. In contrast, metabolic abnormalities linked to chronic kidney illness may worsen oral disorders. It is crucial to include oral health evaluation into the comprehensive care of patients with chronic kidney disease (CKD) due to this intricate connection [7]. Periodontal disease is more common in those with chronic kidney disease (CKD) compared to the general population, according to studies. People who receive dialysis treatment often have severe gum inflammation, tooth loss and periodontal disease. The quality of life might decline because to the discomfort, difficulty chewing, decreased nutrition and psychological suffering caused by several oral disorders [8]. Along with chronic kidney disease (CKD), xerostomia is a common mouth symptom, especially for patients on hemodialysis or antihypertensive drugs. In addition to making, you feel bad; a decrease in saliva production makes you more likely to have cavities, infections and sores on your mouth and gums. The emotional and everyday functioning of patients might be negatively impacted when chronic mouth pain prevents them from speaking, eating and sleeping [9]. There has been less investigation into how the development of chronic kidney disease affects quality of life as it pertains to oral health, despite the growing body of evidence linking dental and systemic health. Few studies have studied OHRQoL across various stages of CKD; most have focused on oral symptoms in dialysis patients. If we want to improve holistic patient treatment and find ways to avoid diseases, we need to know how people perceive their oral health as the condition progresses [10]. Therefore, it is of interest to compare OHRQoL among chronic renal disease patients at different stages of the illness since there is a lack of data on this topic across different stages of CKD and to better understand the significance of interdisciplinary care for patients with CKD, this research seeks to shed light on the correlation between the severity of the disease and the effect of poor dental health on everyday functioning.

## Materials and Methodology:

## Study design and setting:

Patients with chronic kidney disease (CKD) at various stages of disease development were assessed for their oral health-related quality of life (OHRQoL) in this hospital-based cross-sectional comparative research. The research was conducted at a tertiary care teaching hospital's Department of Dentistry in conjunction with the Department of Nephrology. After receiving institutional ethical clearance, data collection was carried out over a six-month period.

## Study population:

One hundred individuals with chronic renal disease who were either hospitalized to the hospital or saw the nephrology outpatient department were a part of the test. Following the acquisition of informed written permission, patients were recruited by a convenience sample approach. Subjects were divided into three categories according to the presence or absence of kidney disease:

The estimated glomerular filtration rate (eGFR) is used to define the KDIGO classification:

[1] Group I - Early stage CKD (Stages 1 and 2)

[2] Group II - Moderate CKD (Stage 3)

[3] Group III - Advanced CKD (Stages 4 and 5)

The treating nephrologist used each participant's most current medical diagnosis and test results to divide them into groups.

## Inclusion criteria:

[1] Patients aged 18 years and above diagnosed with chronic kidney disease.

[2] Patients categorized into CKD stages 1-5 based on eGFR values.

[3] Patients who were able to understand and respond to the questionnaire.

[4] Patients who provided written informed consent to participate in the study. 

## Exclusion criteria:

[1] Patients with acute kidney injury or other temporary renal conditions.

[2] Patients with systemic diseases affecting oral health independently, such as uncontrolled diabetes mellitus or malignancies.

[3] Patients who had undergone renal transplantation.

[4] Patients who were unable to complete the questionnaire due to cognitive or psychological limitations.

## Data collection procedure:

Data collection involved two components: A structured questionnaire and a clinical oral examination.

[1] Socio-demographic and clinical data: Questionnaires and medical records were used to gather data on patients' ages, genders, CKD durations, disease stages, dialysis statuses and medical histories.

[2] Assessment of oral health-related quality of life: To measure quality of life as it pertains to oral health, the Oral Health Impact Profile-14 (OHIP-14) questionnaire was administered. Comprised of fourteen items across seven domains, the OHIP-14 is an instrument that has been extensively verified. It includes:

[1] Functional limitation

[2] Physical pain

[3] Psychological discomfort

[4] Physical disability

[5] Psychological disability

[6] Social disability

[7] Handicap

## Each question was scored using a 5-point Likert scale:

[1] 0 - Never

[2] 1 - Hardly ever

[3] 2 - Occasionally

[4] 3 - Fairly often 

[5] 4 - Very often

Higher overall OHIP-14 scores indicated worse oral health-related quality of life, which might range from 0 to 56.

## Clinical oral examination:

A skilled dentist used sterilized mouth mirrors and explorers in a well-lit environment to do a thorough oral checkup. The following parameters were recorded during the examination:

[1] Dental caries status using the Decayed, Missing and Filled Teeth (DMFT) index.

[2] Periodontal condition assessed using the Community Periodontal Index (CPI).

[3] Presence of xerostomia, oral mucosal lesions, or candidiasis.

[4] Gingival inflammation and bleeding on probing.

[5] Standard infection control protocols were followed during all clinical examinations.

## Sample size:

In order to conduct preliminary comparison analysis across disease stages, a sample size of 100 CKD patients was deemed sufficient. Based on patient availability during the trial period, participants were allocated equally across CKD stages.

## Statistical analysis:

After data collection was complete, it was imported into Excel and analyzed using SPSS 25.0, a statistical package for social sciences. A summary of the study population's demographic and clinical features was provided via descriptive statistics. Mean ± standard deviation was used to represent continuous data like OHIP-14 scores, while frequency and % were used to express categorical variables. The OHRQoL ratings were compared across various phases of CKD using one-way analysis of variance (ANOVA) and, if needed, post-hoc testing. With the use of the Chi-square test, we looked for correlations between several oral health characteristics and the stages of CKD. It was deemed statistically significant if the p-value was less than 0.05.

## Results:

One hundred individuals with a history of chronic kidney disease (CKD) were included in the research. Based on the estimated glomerular filtration rate (eGFR), participants were divided into three groups according to the stages of chronic kidney disease (CKD). Stages 1-2 of early-stage chronic kidney disease (CKD) were included in Group I, Stage 3 in Group II and Stages 4-5 in Group III. Results from clinical oral examinations were documented and participants' oral health-related quality of life was evaluated using the OHIP-14 questionnaire. The breakdown of patients by stage of chronic renal disease in show in (Table 1). Thirty percent were in the early stages (Stages 1-2), 35 percent were in the intermediate stage (Stage 3) and 35 percent were in the advanced stage (Stages 4-5) out of the one hundred people who participated in the research. Quality of life as it relates to oral health was able to be effectively compared across illness stages because to this well balanced distribution. The correlation between chronic kidney disease (CKD) stage and quality of life as it pertains to dental health is seen in (Table 2). Deterioration in oral health-related quality of life was shown by the increasing mean OHIP-14 score as CKD stage progressed. With a relatively low mean OHIP score of 12.4 ± 4.2; only 23.3% of patients in the early stages of CKD reported poor OHRQoL. On the other hand, a higher mean OHIP score of 18.6 ± 5.1 was associated with 42.9% of patients in Stage 3 CKD who reported poor OHRQoL. A mean OHIP score of 26.9 ± 6.3 and a prevalence of 68.6% poor OHRQoL were indicative of the most severe impairment in individuals with advanced CKD. According to the statistically extremely significant difference in OHIP-14 ratings across CKD stages (p<0.001), the severity of renal disease significantly affects the quality of life connected to oral health. Table 3 displays the prevalence of oral health issues among individuals with CKD at various stages. As a disease progressed, the frequency of oral disorders rose sharply. The presence of dental caries increased significantly with disease severity, with 36.7% of early-stage patients, 48.6% of moderate-stage patients and 71.4% of advanced-stage patients showing it (p=0.003). Periodontal disease also has shown a substantial increase, going from 30.0% in early CKD to 77.1% in advanced CKD, with a p-value of less than 0.001. There is a high correlation between decreased salivary flow and the advancement of renal disease (p=0.002), as 20.0% of patients in early CKD, 37.1% in intermediate CKD and 62.9% in advanced CKD reported xerostomia. In addition, 40.0% of patients in advanced CKD had oral mucosal lesions, while only 10.0% of patients in early CKD had them. Patients with advanced kidney disease have a worse oral health-related quality of life and this decline is increasingly noticeable as chronic kidney disease (CKD) advances, according to these results.

## Discussion:

Patients with chronic kidney disease (CKD) at various stages of disease development were assessed for their oral health-related quality of life (OHRQoL) in this research. Results showed a strong correlation between the severity of CKD and worsening oral health and worse OHRQoL. The OHIP-14 scores were higher and the frequency of oral disorders such periodontal disease, dental caries, xerostomia and mucosal lesions were higher in patients with advanced stages of CKD. The significance of include dental healthcare in the all-encompassing treatment of CKD patients is highlighted by these results [11]. Among the patients surveyed, 68.6% reported a poor OHRQoL in advanced CKD stages, compared to 42.9% in moderate stages and 23.3% in early stages. As renal function decreases, the load of oral symptoms grows, as shown by the correlation between CKD severity and a rise in OHIP-14 scores. The average OHIP-14 score in a cross-sectional study of 104 individuals with chronic kidney disease was 14.82 ± 5.86 and there was a significant association between the scores and the stage of renal disease and oral health status (p<0.05). Worsening dental health and worse ability to eat, talk and engage socially may be associated with impaired kidney function [1]. Periodontal disease frequency rose from 30% in early CKD stages to 77.1% in later stages, according to the current research. Systemic inflammation, immunological suppression and poor dental hygiene associated with chronic illness are frequent causes of periodontal inflammation in individuals with chronic kidney disease (CKD). More than 70% of CKD patients had periodontal disease, according to previous studies. This condition is strongly linked to higher OHIP-14 scores and a worse quality of life. Periodontal disease may aggravate the physical and mental components of oral health by influencing chewing, producing discomfort and bleeding gums and adding to tooth mobility and loss [13, 14]. Another prevalent oral ailment found in this research was dental caries, which affected 71.4% of patients in later stages of CKD compared to 36.7% in early stages. Dental caries is more common in persons with chronic kidney disease (CKD) and is associated with poor oral hygiene, dietary changes and decreased salivary flow. Untreated dental illness may considerably impact oral health-related quality of life, according to studies assessing oral health among renal patients [9, 15]. Higher DMFT scores are strongly connected with OHIP-14 scores. In this research, xerostomia was also often reported by CKD patients; in fact, 62.9% of patients in the later stages experienced symptoms of dry mouth. Fluid limitations, metabolic abnormalities and long-term medication usage, particularly of antihypertensive and diuretic medications, may all lead to reduced salivary flow in CKD. Some symptoms of xerostomia include burning, changes in how you perceive taste, trouble chewing and swallowing and an increased risk of dental cavities and infections of the mouth. Research on dialysis patients has shown that dry mouth and discomfort are major factors in poor overall health-related quality of life (OHRQoL), impacting dimensions such as physical pain and psychological discomfort [16]. As an additional point, the prevalence of oral mucosal lesions was 10% in early stages of CKD but 40% in later stages, a significant increase. The buildup of uremic toxins, changes in immunological function, or dietary deficits may all lead to these lesions. Pain and trouble eating may be caused by oral mucosal abnormalities such candidiasis, ulcerations and uremic stomatitis. This can further influence the patient's nutritional intake and overall wellness [11]. Chronic periodontal infection is linked with a systemic inflammatory load, which is another crucial component determining OHRQoL in CKD patients. Multiple studies have pointed to a two-way street between periodontal disease and chronic kidney disease (CKD), with inflammation in the gums potentially exacerbating inflammation in the body and hastening the course of the illness. Despite a high burden of oral disease, patients with chronic kidney disease demonstrate a paradoxically positive oral health-related quality of life, with impairments mainly linked to pain and psychological factors, highlighting the need for integrated dental care in CKD management [17]. Psychological stress and low self-esteem are common among chronic kidney disease (CKD) patients because of the disease itself, the frequency of hospital visits and the dietary restrictions that patients must adhere to. Social connections and emotional wellness may be further impacted by oral health issues such bad breath, tooth loss and trouble speaking. Consequently, people with severe CKD report that oral disorders have a much greater effect on their quality of life [15]. Poor dental health is linked to poorer systemic results, such as an increased risk of infection and death, according to studies analyzing individuals receiving peritoneal dialysis. In order to enhance systemic and oral health, our results stress the need of CKD patients treating dental issues and keeping up with proper oral hygiene [13]. The current study's results corroborate those of earlier studies showing that people with chronic kidney disease (CKD) have a lower quality of life when it comes to their oral health compared to healthy people. This impairment is especially noticeable in patients who are on dialysis or have advanced stages of renal disease. Consequently, CKD treatment should include preventative dental care and screenings on a regular basis.

## Conclusion:

Quality of life as it relates to dental health was shown to be significantly associated with the advancement of chronic kidney disease in the current investigation. Higher OHIP-14 scores and worse quality of life were associated with periodontal disease, dental caries, xerostomia and oral mucosal lesions in patients with advanced stages of chronic kidney disease. The need of early dental evaluation, preventative oral care and interdisciplinary teamwork between dentistry and nephrologists is emphasized by these results. Patients' general health and the incidence of oral problems may both benefit from oral healthcare's incorporation into standard CKD therapy.

## Figures and Tables

**Table 1:** Distribution of study participants according to CKD stage

**CKD Stage**	**Number (n)**	**Percentage (%)**
Early Stage (Stages 1-2)	30	30%
Moderate Stage (Stage 3)	35	35%
Advanced Stage (Stages 4-5)	35	35%
Total	100	100%

**Table 2:** Comparison of Mean OHIP-14 Scores among CKD Stages

**CKD Stage**	**Mean OHIP-14 Score± SD**	**Poor OHRQoL n (%)**
Early Stage (Stages 1-2)	12.4± 4.2	7 (23.3%)
Moderate Stage (Stage 3)	18.6± 5.1	15 (42.9%)
Advanced Stage (Stages 4-5)	26.9± 6.3	24 (68.6%)
p-value	<0.001	

**Table 3:** Distribution of oral health conditions among CKD Stages

**Oral Condition**	**Early CKD (n=30)**	**Moderate CKD (n=35)**	**Advanced CKD (n=35)**	**p-value**
Dental Caries	11 (36.7%)	17 (48.6%)	25 (71.4%)	0.003
Periodontal Disease	9 (30.0%)	18 (51.4%)	27 (77.1%)	<0.001
Xerostomia	6 (20.0%)	13 (37.1%)	22 (62.9%)	0.002
Oral Mucosal Lesions	3 (10.0%)	7 (20.0%)	14 (40.0%)	0.011
